# Supplementary material for: Reconstruction and normalization of LISA for spatial analysis
Source: PLoS One. 2024 May 22;19(5):e0303456. doi: 10.1371/journal.pone.0303456 (PMC11111027; doi:10.1371/journal.pone.0303456)
Supplement: S1 File — This is a microcosm of Anselin’s paper on LISA. The key parts of Anselin’s mathematical reasoning are extracted, and the main errors in the reasoning process are revealed. This file uses Anselin’s original symbol system. Through this file, readers can more easily grasp the essence of the problem. (DOCX) [file pone.0303456.s001.docx]

## Appendix 1: Anselin’s derivation and expressions for LISA

LISA proposed by Dr. Anselin (1995) in one of his excellent papers is an important local measure set for geographical spatial analysis. However, during my teaching process of quantitative geography, I discovered a series of oversights or even errors in Anselin’s seminal paper on LISA. Due to Anselin's significant academic influence, these omissions and errors have been spreading through literature, and fallacies have been passed down to this day. Because of the logical contradiction between the calculation results and theoretical expectations, I have to expend energy every year to repeatedly explain to students. Therefore, I have decided to write a paper to systematically clarify Anselin's mistakes and provide standardized LISA expressions in order to benefit more readers. After all, scientists respect authority, but science itself respects logic and facts more.

### A1.1. Basic requirements

In Anselin’s seminal paper, he defined two general requirements for a local indicator of spatial association (LISA). The basic requirements are as below: “a. the LISA for each observation gives an indication of the extent of significant spatial clustering of similar values around that observation. b. the sum of LISAs for all observations is proportional to a global indicator of spatial association.” For a statistic *L_i_* based on a variable *y_i_* observed at location *i*, the second requirement of a LISA, may be stated formally as

$\sum_{i=1}^{n} L_{i}=\gamma\Lambda$, (A1)

where *Λ* is a global indicator of spatial association and *γ* is a scale factor. Unfortunately, based on row-normalized spatial weights matrix, the second requirement cannot be really satisfied in both theoretical derivation and empirical analyses.

The following reasoning process is adapted from Anselin's original paper. For easy understanding, I completely adopt his symbols, but one or more concepts will be changed. For example, row standardization is replaced by row normalization (Table A1).

**Table A1 The symbol system of variables and weights in Anselin’s seminal paper**

| Measure | Method | Calculation formula | Property |
| --- | --- | --- | --- |
| Size variable | Centralization | $z_{i}=y_{i}-\bar{y}$, $z_{j}=y_{j}-\bar{y}$ | The mean value is 0 |
|  | Standardization by *z*-score | $z_{i}=\frac{y_{i}-\bar{y}}{\sqrt{m_{2}}}$, $z_{j}=\frac{y_{j}-\bar{y}}{\sqrt{m_{2}}}$ | The mean value is 0 and the standard deviation is 1 |
| Weight | Global normalization | $\frac{w_{ij}}{S_{0}}=w_{ij}/\sum_{i=1}^{n} \sum_{j=1}^{n} w_{ij}$ | The sum of weights equals 1 |
|  | No normalization | $w_{ij}$ | The sum of weights depends |
|  | Row normalization | $\frac{w_{ij}}{w_{i}}=w_{ij}/\sum_{j=1}^{n} w_{ij}$ | The sum of weights equals *n* |

**Note**: According to Anselin (1995), “the weights *w_ij_* may be in row-standardized form, though this is not necessary, and by convention, *w_ii_*=0.” This suggests, both no normalization weights and row normalization weights are acceptable for calculating LISAs. For a variable *y_i_* observed at location *i*, the mean is represented by *ȳ*.

### A1.2. Local Moran’s index

A local Moran statistic for an observation *i* may be defined as

$I_{i}=z_{i}\sum_{j=1}^{n} w_{ij}z_{j}$, (A2)

where *z_i_* or *z_j_* is centralized variable, *w_ij_* denotes weights, which may be in row-standardized form or not, though this is not necessary. The sum of local Moran’s *I* is

$\sum_{i=1}^{n} I_{i}=\sum_{i=1}^{n} z_{i}\sum_{j=1}^{n} w_{ij}z_{j}$. (A3)

So for the global indicator, Morn’s *I* is

$I=\frac{n}{S_{0}}\frac{\sum_{i=1}^{n} \sum_{j=1}^{n} w_{ij}(x_{i}-\bar{x})(x_{j}-\bar{x})}{\sum_{i=1}^{n} (x_{i}-\bar{x})^{2}}=\frac{n}{S_{0}}\frac{\sum_{i=1}^{n} \sum_{j=1}^{n} w_{ij}z_{i}z_{j}}{\sum_{i=1}^{n} z_{i}^{2}}=\frac{\sum_{i=1}^{n} I_{i}}{S_{0}(\frac{1}{n}\sum_{i=1}^{n} z_{i}^{2})}=\frac{1}{S_{0}m_{2}}\sum_{i=1}^{n} I_{i}$, (A4)

where

$S_{0}=\sum_{i=1}^{n} \sum_{j=1}^{n} w_{ij}$ (A5)

is the sum of the weights, and

$m_{2}=\frac{1}{n}\sum_{i=1}^{n} z_{i}^{2}$ (A6)

denotes the second moment, a consistent, but not unbiased estimate of the population variance. The factor of proportionality between the sum of the local and the global Moran index is

$\gamma=S_{0}m_{2}$. (A7)

Please note that equation (A4) is based on global normalization weights. This is the necessary condition to guarantee the validness of equations (A8), (A10), and (A11) given later. Equation (A4) can be expressed as

$\sum_{i=1}^{n} I_{i}=S_{0}m_{2}I=\gamma I$. (A8)

Note that for a row-standardized spatial weights matrix, *S*_0_=*n*, so that

$\gamma=S_{0}\frac{1}{n}\sum_{i=1}^{n} z_{i}^{2}=\sum_{i=1}^{n} z_{i}^{2}$. (A9)

And for the standardized variable based on *z*-score, *m*_2_=1, so that

$\gamma=S_{0}m_{2}=S_{0}=n$. (A10)

Therefore, for the row-standardized spatial weights matrix, equation (A8) can be written as

$\sum_{i=1}^{n} I_{i}=nI$. (A11)

The local Moran indexes would then be computed as

$I_{i}=\frac{z_{i}}{m_{2}}\sum_{j=1}^{n} w_{ij}z_{j}$, (A12)

which is actually a local Moran’s *I* based on *z*-score of observations *y_i_*.

Formally, there seems to be no problem with the above mathematical process. Based on globally normalized spatial weight matrix, equations (A7), (A8), and (A11) have no problem. However, based on row-normalization weights matrix, equations (A4), (A7), (A8), and (A11), are not correct. The normalized weight matrix by sum differs from the normalized weight matrix by row in mathematical structure. With the help of simple mathematical experiments, it can be found that Anselin’s LISAs based on row-normalization weights cannot satisfy his second requirement, which specified by equation (A1). Let’s see the following mathematical process. The row sum of the weights is

$w_{i}=\sum_{j=1}^{n} w_{ij}$. (A13)

Summing equation (A13) yields

$\sum_{i=1}^{n} w_{i}=\sum_{i=1}^{n} \sum_{j=1}^{n} w_{ij}=S{}_{0}$. (A14)

However, the weights based on row normalization is as follows

$w_{ij}^{*}=\frac{w_{ij}}{w_{i}}=w_{ij}/\sum_{j=1}^{n} w_{ij}$. (A15)

Double summing equation (A15) yields

$\sum_{i=1}^{n} \sum_{j=1}^{n} w_{ij}^{*}=\sum_{i=1}^{n} (\frac{1}{w_{i}}\sum_{j=1}^{n} w_{ij})=\sum_{i=1}^{n} (1)=n$. (A16)

No problem can be found equations (A14) and (A16), which is deceiving. The local Moran’s indexes based on row-normalization weights is

$I_{i}=\frac{z_{i}}{\sum_{j=1}^{n} w_{ij}}\sum_{j=1}^{n} w_{ij}z_{j}=\frac{z_{i}}{w_{i}}\sum_{j=1}^{n} w_{ij}z_{j}=z_{i}\sum_{j=1}^{n} \frac{w_{ij}}{w_{i}}z_{j}=z_{i}\sum_{j=1}^{n} w_{ij}^{*}z_{j}$. (A17)

Summing equation (A17) yields

$\sum_{i=1}^{n} I_{i}=\sum_{i=1}^{n} \frac{z_{i}}{\sum_{j=1}^{n} w_{ij}}\sum_{j=1}^{n} w_{ij}z_{j}=\sum_{i=1}^{n} z_{i}\sum_{j=1}^{n} \frac{w_{ij}}{w_{i}}z_{j}=\sum_{i=1}^{n} \frac{z_{i}}{w_{i}}\sum_{j=1}^{n} w_{ij}z_{j}\neq\gamma I$. (A18)

We can never derive a relation similar to equation (A8), which satisfies equation (A1).

### A1.3. Local Geary’s coefficient

Using the same principles as before, a local Geary statistic based on no normalized weights and no standardized variable for each observation I was defined

$c_{i}=\sum_{j=1}^{n} w_{ij}(z_{i}-z_{j})^{2}$. (A19)

Based on standardized variable, the local Geary coefficient was expressed as

$c_{i}=\frac{1}{m_{2}}\sum_{j=1}^{n} w_{ij}(z_{i}-z_{j})^{2}$. (A20)

The notation is the same as before. Without loss of generality, the summation of the *c_i_* over all observations is

$\sum_{i=1}^{n} c_{i}=\frac{1}{m_{2}}\sum_{i=1}^{n} \sum_{j=1}^{n} w_{ij}(z_{i}-z_{j})^{2}=n\sum_{i=1}^{n} \sum_{j=1}^{n} w_{ij}(z_{i}-z_{j})^{2}/\sum_{i=1}^{n} z_{l}^{2}$. (A21)

In comparison, the global Geary statistic is

$c=\frac{n-1}{2S_{0}}\sum_{i=1}^{n} \sum_{j=1}^{n} w_{ij}(z_{i}-z_{j})^{2}/\sum_{i=1}^{n} z_{l}^{2}$. (A22)

Substituting equation (A22) into equation (A21) yields

$\sum_{i=1}^{n} c_{i}=\frac{2nS_{0}}{n-1}c$. (A23)

Comparing equation (A23) into equation (A1) indicates that the factor of proportionality between the sum of the local and global Geary statistics is

$\gamma=\frac{2nS_{0}}{n-1}$. (A24)

Formally, for row-normalized weights, *S*_0_=*n*; therefore, the proportionality factor is *γ*=2*n*^2^/(*n*-1).

On the surface, there is no problem with the above mathematical reasoning process. In fact, there is a bug. The row normalized weights was unintentionally replaced by the global normalized weights in the derivation. Based on row normalized weights and standardized variable, the local Geary coefficient is actually as below

$c_{i}=\frac{1}{m_{2}w_{i}}\sum_{j=1}^{n} w_{ij}(z_{i}-z_{j})^{2}=\frac{1}{m_{2}}\sum_{j=1}^{n} w_{ij}(z_{i}-z_{j})^{2}/\sum_{j=1}^{n} w_{ij}$. (A25)

From equation (A25) it follows

$\sum_{i=1}^{n} c_{i}=n\sum_{i=1}^{n} \frac{1}{w_{i}}\sum_{j=1}^{n} w_{ij}(z_{i}-z_{j})^{2}/\sum_{j=1}^{n} z_{i}^{2}\neq\gamma c$. (B26)

which cannot satisfy the second requirement defined by Anselin (1995).

### A1.4. Conclusions

If you find the derivation process above confusing, you can use MS Excel to conduct a mathematical experiment with a simple example. Both Anselin's mathematical reasoning and the author's mathematical reasoning can be verified step by step through concise cases. In short, Anselin’s paper on LISA involves three issues that need to be addressed. First, *there is a mistake of mathematical reasoning resulted from step skip of mathematical transformation*. This is an unintentional mistake, but it leads readers to misunderstand the relationship between global normalized spatial weight matrix and row-normalized (row-standardized) spatial weight matrix. Second, *the row-normalized spatial weight matrix violates the distance axiom*. A spatial weight matrix is based on distance matrix or generalized distance matrix, which must conforms to distance axiom. Otherwise, the calculation result of the global or local Moran’s *I* may appear abnormal. Third, *the basic difference between Moran’s I and Geary’s C was omitted*. Moran’s *I* is based on spatial population, while Geary’s *C* is based on spatial sample. Different definitions lead to different application directions. However, in the definitions of LISA, the local Geary’s *C* is based on spatial population rather spatial sample.

**[Reference]** Anselin L (1995). Local indicators of spatial association—LISA. *Geographical Analysis*, 27(2): 93–115
