# Supplementary material for: Reconstruction and normalization of LISA for spatial analysis
Source: PLoS One. 2024 May 22;19(5):e0303456. doi: 10.1371/journal.pone.0303456 (PMC11111027; doi:10.1371/journal.pone.0303456)
Supplement: S2 File — This file show common concepts and methods of value transformation and corresponding formulae for variable standardization. This document clarifies some confusion and inappropriate expressions regarding variable standardization in the literature. (DOCX) [file pone.0303456.s002.docx]

## Appendix 2: Value transformation methods and formulae

It is necessary to use appropriate value transformations in order to effectively utilize a mathematical method. In time series analysis, the most basic and commonly used value transformation method is termed variable *centralization*, which means subtracting the mean value of a variable from it. Centralization can simplify temporal autoregressive models and make spectral analysis results more intuitive. Many methods of spatial analysis in geography were initially obtained through analogy with time series analysis methods, thus inheriting the tradition of variable centralization in time series analysis.

If the purpose of a value transformation is to reduce or even eliminate the influence of variable dimensionality, then the value transformation method belongs to generalized variable *standardization*. General standardization includes *narrow standardization* and *normalization*. Narrowly defined standardization is based on variable centralization, characterized by a mean of 0 after value transformation. In contrast, normalization is to make the value of a variable fall between 0 and 1. Various value transformation methods and corresponding formulae are tabulated as follows for reference (Table B).

**Table B Value transformation methods commonly used in mathematical modeling and quantitative analysis**

| Type | Subtype | Term | Classification in SPSS | Formula |
| --- | --- | --- | --- | --- |
| Centralization | N/A | N/A | N/A (mean of 0) | $y_{i}=x_{i}-\bar{x}$ |
| Generalized standardization | Narrow standardization (mean of 0) | *Z* scores | *Z* scores | $\begin{matrix} z_{i}=\frac{y_{i}}{\sigma} \\ =\frac{x_{i}-\bar{x}}{\sigma} \end{matrix}$ |
|  |  | Range standardization | Range -1 to 1 | $z_{i}=\frac{x_{i}-\bar{x}}{x_{\max}-x_{\min}}$ |
|  | Normalization (range 0 to 1) | Range normalization | Range 0 to 1 | $z_{i}=\frac{x_{i}-x_{\min}}{x_{\max}-x_{\min}}$ |
|  |  | Sum normalization | N/A (sum of 1) | $y_{i}=\frac{x_{i}}{\sum_{i=1}^{n} x_{i}}=\frac{x_{i}}{n\bar{x}}$ |
|  | Others | N/A | Mean of 1 | $y_{i}=\frac{x_{i}}{\bar{x}}$ |
|  |  | Precision weighting | Standard deviation of 1 | $y_{i}=\frac{x_{i}}{\sigma}$ |
|  |  | N/A | Maximum magnitude of 1 | $y_{i}=\frac{x_{i}}{x_{\max}}$ |

**Note:** For example, in order to effectively carry out cluster analysis, it is necessary to standardize variables in a broad sense in in statistical analysis software IBM SPSS. SPSS bears various functions for generalized standardization of variables except for sum-based normalization. (2) Suppose that sample size is *n*. The meanings of symbols in the table are as follows: *x* is original variable, *x*_min_ denotes the minimum value, *x*_max_ denotes the maximum value, *y* refers to a primary transformation variable, and *z* represents a secondary transformation variable. As for the statistics, *x*-bar denotes mean value, and *σ* refers to standard deviation.
