# Supplementary material for: Reconstruction and normalization of LISA for spatial analysis
Source: PLoS One. 2024 May 22;19(5):e0303456. doi: 10.1371/journal.pone.0303456 (PMC11111027; doi:10.1371/journal.pone.0303456)
Supplement: Attachment — Submitted filename: Response to Academic Editor and Reviewer 2024-03-15.docx [file pone.0303456.s006.docx]

## Response to reviewers

### Reply to Academic Editor (AE)

Additional Editor Comments:

**[AE]** The authors conduct a series of rigorous mathematical reasoning of LISA showing that using row-normalized spatial weight matrix would violate the second basic requirement for LISA. As stated by the authors, this is not substantial innovation, but it is helpful in figuring the logic of local spatial autocorrelation statistics. I have some minor comments.

**[Reply]** During my teaching of quantitative geography, I discovered a series of oversights or even errors in Anselin's paper on LISA. Due to Anselin's significant academic influence, these omissions and errors have been spreading through literature, and fallacies have been passed down to this day.

Because of the logical contradiction between the calculation results and theoretical expectations, I have to expend energy every year to repeatedly explain to students. Therefore, I have decided to write this article to systematically clarify Anselin's mistakes and provide standardized LISA expressions in order to benefit more readers. After all, scientists respect authority, but science itself respects logic and facts more.

**[AE]**1. Page 4. The spatial contiguity matrix V is not explained in detail. There are many definitions of spatial contiguity matrix, and would Rook, Queen, or distance-based matrix have any difference on the calculation of LISA?

**[Reply]** Now, a paragraph has been added to the text to discuss the different definitions of spatial contiguity matrix as follows:

“Finally, it is appropriate to briefly discuss the definition of spatial weight matrix. Spatial autocorrelation analysis depends on spatial contiguity matrix, which has multiple definitions. In fact, definition of spatial contiguity involves different spatial effects. Spatial effects of geographical processes fall into two categories: *action at a distance* and *local action* (Chen *et al*, 2021). Local action can be expressed with step function in mathematics and nominal variable in value. In spatial autocorrelation analysis, the spatial contiguity matrix based on local action is mainly applicable to relationships between regions. The spatial contiguity relationship of regions bears three ways of definitions, that is, Rook’s contiguity, Bishop’s contiguity, and Queen’s contiguity (Widip *et al*, 2013). Rook’s contiguity plus Bishop’s contiguity yields Queen’s contiguity. In fact, Rook’s contiguity corresponds to von Neumann’s neighborhood definition, while Queen’s contiguity corresponds to Moore’s neighborhood definition (Batty *et al*, 1997). Action at a distance can be reflected by certain distance, including Euclidean distance, travel time, transportation mileage and so on. When converting distances into spatial contiguity matrix, a certain spatial contiguity function needs to be adopted. Common spatial contiguity functions include absolute step function, relative step function, exponential function, and distance inverse function (a type of hyperbolic function) (Chen, 2012; Haggett *et al*, 1977; Ordland, 1988). Distance-based spatial contiguity matrix is suitable for networks of locations such as urban systems. In this case, based on the step function, spatial contiguity is represented by nominal variable (dummy variable in discrete format); based on other functions, the spatial contiguity is represented by metric variable (continuous variable). Although the function expressions are different, the logic behind them is consistent with one another. Mathematics is the pinnacle of logic. In mathematics, the most basic function is exponential function. Various forms of simple functions can be reduced to exponential function. The step function is an extreme form of an exponential function, and moving average on the step function can yield an inverse distance function (Chen, 2015). So, using different functions to define spatial contiguity matrices will definitely affect the calculation results, but it has no impact on the mathematical reasoning results and the logical relationships behind them. The reason why row normalization weight matrix affects mathematical reasoning results is because the logic behind the spatial weight matrix has been changed, and the logic is regulated by the distance axiom. Scientific research typically involves three worlds: the *real world*, the *mathematical world*, and the *computational world* (Casti, 1996). The process of mathematical transformation and derivation belongs to the mathematical world, while the selection of spatial weight matrix forms belongs to the computational world. The key is to choose the appropriate spatial contiguity matrix definition method for different geographic systems based on different situations (Chen, 2012). ”

- Batty M, Couclelis H, Eichen M (1997). Editorial: Urban systems as cellular automata. *Environment and Planning B: Planning and Design*, 24: 159-164
- Casti JL (1996). *Would-Be Worlds: How Simulation Is Changing the Frontiers of Science*. New York: John Wiley and Sons
- Chen YG (2015). Power-law distributions based on exponential distributions: Latent scaling, spurious Zipf’s law, and fractal rabbits. *Fractals*, 23(2): 1550009
- Chen YG, Li YJ, Feng S, Man XM, Long YQ (2021). Gravitational scaling analysis on spatial diffusion of COVID-19 in Hubei province, China. *PLoS ONE*, 16(6): e[0252889](https://doi.org/10.1371/journal.pone.0252889)
- Widip CA, Utomo WH, Yulianto SJP (2013). Identification of spatial patterns of food insecurity regions using Moran’s I (Case study: Boyolali regency). *International Journal of Computer Applications*, 72(2): 54-62

**[AE]** 2. Page 4. Is it necessary to stress that i≠j in Equation (1)?

**[Reply]** Yes. A sentence is added to the text as follows: “In equation (1), *i*≠*j*, otherwise *v_ij_*=0.”

**[AE]** 3. Page 4 to Page 5. It might be that I misunderstand something. The z-score normalization is only divided by σ, what is the meaning of σ. It seems that σ is calculate by x. But if we treat x_i-x ̅ as a whole, the normalization should be based on the σ of x_i-x ̅. Compared with equation 1, dividing only by σ. could not be called z-score normalization.

**[Reply]** It should be z-score standardization. No z-score normalization. Where value transformation is concerned, normalization belongs to standardization in a broad sense. The purpose of standardization is to reduce or even remove the influence of variable dimensionality. Normalization is to make the values of a variable fall between 0 and 1. Normalization can be divided into two types: range based normalization and sum based normalization. Now, an appendix is added to clarify the relationships between different methods of value transformation in multivariable statistical analysis (Appendix 2).

Suppose that the original variable is *x*. The formula of variable centralization is as follows

$y_{i}=x_{i}-\bar{x}$;

Thus, the mean value of the converted variable is 0 (Mean of 0).

The formula of precision weighting variable is as follows

$y_{i}=\frac{x_{i}}{\sigma}$;

Thus, the standard deviation of the converted variable is 1 (Standard deviation of 1).

The formula of *z*-score standardization of a variable is as follows

$z_{i}=\frac{y_{i}}{\sigma}=\frac{x_{i}-\bar{x}}{\sigma}$.

As can be seen, centralization + precision weighting = *z*-score (Mean of 0, Standard deviation of 1).

The formula of variable normalization by range is as follows

$z_{i}=\frac{x_{i}-x_{\min}}{x_{\max}-x_{\min}}$, $z_{i}=\frac{x_{\max}-x_{i}}{x_{\max}-x_{\min}}$.

As a result, the values of the variable fall between 0 and 1.

The formula of variable normalization by sum is as follows

$y_{i}=\frac{x_{i}}{\sum_{i=1}^{n} x_{i}}=\frac{x_{i}}{n\bar{x}}$.

As a result, the values of the variable fall between 0 and 1 and the sum of variable values is 1.

### Appendix 2: Value transformation methods anformulae

It is necessary to use appropriate value transformations in order to effectively utilize a mathematical method. In time series analysis, the most basic and commonly used value transformation method is termed variable *centralization*, which means subtracting the mean value of a variable from it. Centralization can simplify temporal autoregressive models and make spectral analysis results more intuitive. Many methods of spatial analysis in geography were initially obtained through analogy with time series analysis methods, thus inheriting the tradition of variable centralization in time series analysis.

If the purpose of a value transformation is to reduce or even eliminate the influence of variable dimensionality, then the value transformation method belongs to generalized variable *standardization*. General standardization includes *narrow standardization* and *normalization*. Narrowly defined standardization is based on variable centralization, characterized by a mean of 0 after value transformation. In contrast, normalization is to make the value of a variable fall between 0 and 1. Various value transformation methods and corresponding formulae are tabulated as follows for reference (Table A).

**Table A Value transformation methods commonly used in mathematical modeling and quantitative analysis**

| Type | Subtype | Term | Classification in SPSS | Formula |
| --- | --- | --- | --- | --- |
| Centralization | N/A | N/A | N/A (mean of 0) | $y_{i}=x_{i}-\bar{x}$ |
| Generalized standardization | Narrow standardization (mean of 0) | *Z* scores | *Z* scores | $\begin{matrix} z_{i}=\frac{y_{i}}{\sigma} \\ =\frac{x_{i}-\bar{x}}{\sigma} \end{matrix}$ |
|  |  | Range standardization | Range -1 to 1 | $z_{i}=\frac{x_{i}-\bar{x}}{x_{\max}-x_{\min}}$ |
|  | Normalization (range 0 to 1) | Range normalization | Range 0 to 1 | $z_{i}=\frac{x_{i}-x_{\min}}{x_{\max}-x_{\min}}$ |
|  |  | Sum normalization | N/A (sum of 1) | $y_{i}=\frac{x_{i}}{\sum_{i=1}^{n} x_{i}}=\frac{x_{i}}{n\bar{x}}$ |
|  | Others | N/A | Mean of 1 | $y_{i}=\frac{x_{i}}{\bar{x}}$ |
|  |  | Precision weighting | Standard deviation of 1 | $y_{i}=\frac{x_{i}}{\sigma}$ |
|  |  | N/A | Maximum magnitude of 1 | $y_{i}=\frac{x_{i}}{x_{\max}}$ |

Note: For example, in order to effectively carry out cluster analysis, it is necessary to standardize variables in a broad sense in in statistical analysis software IBM SPSS. SPSS bears various functions for generalized standardization of variables except for sum-based normalization. (2) Suppose that sample size is *n*. The meanings of symbols in the table are as follows: *x* is original variable, *x*_min_ denotes the minimum value, *x*_max_ denotes the maximum value, *y* refers to a primary transformation variable, and *z* represents a secondary transformation variable. As for the statistics, *x*-bar denotes mean value, and *σ* refers to standard deviation.

**[AE]** 4. Page 6 Table 1. What is the benefit of using the normalized weight matrix instead of the original one?

**[Reply]** This is a good question worth discussing.

“In the new framework for LISA, the spatial weight matrix is normalized by sum. This is a type of global normalization in value transformation. There are several benefits to using a globally normalized weight matrix. We know that mathematics is in a sense a science relying highly on form. The same mathematical method often has vastly different effects when expressed in different forms. For spatial autocorrelation, using a normalized spatial weight matrix instead of a non-normalized weight matrix results in at least the following advantages. First, by normalized weight matrix, it is very convenient to calculate the global Moran’s index *I* and local Moran’s indexes *I_i_*, and reflect the clear relationship between the two, *I* and *I_i_* (Chen, 2013). Second, normalizing weight matrix, we can obtain a standardized Moran’s scatterplot, where the slope of the trend line is exactly equal to the global Moran’s index value (Chen, 2023). Third, based on normalized weight matrix, the structure of the parameters of the spatial autoregressive models can be clearly revealed using the spatial autocorrelation coefficients. Fourth, it makes the values of local Moran’s index and local Geary’s coefficient more intuitive. The fourth advantage mentioned above is more relevant to the research in this work. Many basic measures and models of spatial statistical analysis are rooted in conventional statistics and are created by analogy with time series analysis methods. The common measures and models of time series analysis, such as autocorrelation coefficients and autoregressive models, are also rooted in traditional statistical theories. The development of statistics took place in the wider context of the Victorian culture of measurement (Magnello and van Loon, 2009). For simplicity, the numerous data of measurement results are usually condensed into an index (Wheelan, 2013). In this case, an index is often treated as a characteristic measurement (Haggett *et al*, 1977; Taylor, 1983). A good index either has a pair of clear boundary values, a clear critical value, or even a combination of both. Based on standardized variable and globally normalized spatial weight matrix, the values of the local Moran’s indexes fall between -1 and 1, the corresponding critical value is 0; and the values of the local Geary’s coefficient falls between 0 and 2, and the corresponding critical value is 1.”

- Wheelan C (2013). *Naked Statistics: Stripping the Dread from the Data*. New York and London: W. W. Norton & Company

In fact, to a certain extent, many methods and models of spatial analysis are generated through analogy with time series analysis methods. However, there seems to be some misalignment in the analogy between spatial autocorrelation analysis and time series analysis. In time series analysis, temporal autocorrelation coefficient is actually based on standardized variable and globally normalized weight matrix—the time lag parameter can be transformed into weight matrix. Temporal auto-regressive modeling is often based on centralized variable. In spatial statistical analysis, the expression of Moran’s index is usually based on centralized variable rather than standardized variable (Table B).

**Table B Comparison of variable and matrix transformation between time series analysis and spatial statistical analysis (in general)**

| Type | Time series analysis | Spatial statistical analysis |
| --- | --- | --- |
| Autocorrelation | Variable is standardized by *z*-score | Variable can be centralized by mean |
|  | Weight matrix transformed from time lag is normalized by sum | Spatial contiguity matrix is not normalized by sum in many cases |
| Auto-regression/lag-regression | Variable is centralized by mean | No variable transformation in majority of cases |
|  | It is better not to normalize the weight matrix transformed from time lag by sum | No spatial contiguity matrix transformation in many cases |

In multivariate statistical analysis, the value transformation method usually depends on the modeling purpose (Table C).

**Table C The effect of value transformation applied in conventional mathematical methods (simple examples)**

| Item | Modeling purpose | Value transform | Advantages or disadvantages |
| --- | --- | --- | --- |
| Linear regression modeling | For explanation | Variable standardization by *z*-score | In this case, the significance of a variable's influence can be directly reflected by regression coefficient values. |
|  | For prediction | No value transformation | In this case, predicted values can be obtained directly. |
| Frequency-spectrum analysis | For periodicity analysis | Variable centralization by mean | In this case, periodicity can be visually observed. |
|  | For trend analysis | No value transformation | Based on centralized variable, it is generally not possible to fit a trend model to the observed data |

**[AE]** 5. Page 2 In the first paragraph of introduction, “Gravity models, spatial interaction models, and spatial autocorrelation analysis are the main approaches…”, parallel relationship might not be appropriate for gravity model and spatial interaction model since the former is one type of the latter.

**[Reply]** Indeed, the expression of this sentence is not rigorous enough. In order to avoid misunderstanding, I deleted “Gravity models” from the sentence. Now, the sentence is as below:

“Spatial interaction models and spatial autocorrelation analysis are the main approaches to research spatial correlation processes.”

In fact, My initial meaning was as follows: “Classical gravity models, Wilson’s spatial interaction models, and spatial autocorrelation analysis are the main approaches…”.

**[For communication purposes only]** The generalized spatial interaction model includes the gravity model. When we talk about spatial interaction models, we usually first think of Wilson's series of models based on the maximum entropy principle. In the literature, the spatial interaction model is often equated with the gravity model in terms of concept. However, there are differences between classical gravity models and Wilson’s spatial interaction models in deep structure (Chen, 2023). If we want to derive the gravity model from Wilson’s spatial interaction models, we must rely on a hypothesis, that is, there is an allometric scaling relation between spatial flow quantity (e.g., cargo flow) and place size (e.g., city size) (Chen, 2023). A comparison between the classical or improved gravity models and Wilson’s spatial interaction models was once drawn by Chen (2023) as follows (Table B). The results are for reference only.

- Chen YG (2015). The distance-decay function of geographical gravity model: power law or exponential law? *Chaos, Solitons and Fractals*, 77: 174-189
- Chen YG (2023). Demonstration of duality of fractal gravity models by scaling symmetry. *Chaos, Solitons and Fractals*, 170: 113354

**[For reference only] Table B--The similarities and differences between dual gravity models and Wilson’s spatial interaction models**

| Type | Dual gravity models | Wilson’s spatial interaction models |
| --- | --- | --- |
| Theoretical basis | Entropy maximization and allometric scaling law | Entropy maximization and nonlinear programming |
| Geographical world | Real world | Ideal world |
| Distance decay function | Inverse power law | Negative exponential function |
| Spatial effect | Action at a distance | Quasi-locality |
| Autocorrelation function | Tail off | Tail off |
| Partial autocorrelation function | Tail off | Cut off |
| Application direction | Behavioral research and positive analysis | Normative research and predictive analysis |
| Alternative form | Based on negative exponential decay | Based on inverse power-law decay |

**Source**: Chen YG. Demonstration of duality of fractal gravity models by scaling symmetry. *Chaos, Solitons and Fractals*, 2023, 170: 113354

### Reply to Reviewer

**[Reviewer #1]**: The article is an interesting work. Provide clarifications on the local spatial association indicator (LISA) and the local Geary indicator, widely used in the literature. This article aims to reconstruct the calculation formulas of local Moran indices and Geary coefficients through mathematics, presenting corrections or modifications to these indicators. Finally, it presents an application to real data.

**[Reviewer #1]** page 1: significant measures, or important measures

**[Reply]** In order to avoid misunderstandings among readers of the paper, the term "important" has been replaced by "important".

**[Reviewer #1]**page 3: significant measures: the use of the word significant is used for hypothesis testing!

**[Reply]** The word “significant” has been replaced by “important” to avoid understanding from readers.

According to Oxford Dictionary, the word “significant” means “large or important enough to have an effect or to be noticed”. According to Collins Dictionary, “A significant amount or effect is large enough to be important or affect a situation to a noticeable degree.”

In statistics, the words “significance” of “significance testing” means that “According to the given criteria, the calculation result is important enough not to be ignored”. The so-called "given criteria" is the "level of significance", or equivalently, the "confidence level". As we know, confidence level=(1-significance level)*100%.

**[Reviewer #1]** Page 5: equation (2).

**[Reply]** Thanks. The sentence “Dividing equation (1) by *V*_0_ yields spatial weighted auto-covariance as follows” has been corrected as “Dividing equation (2) by *V*_0_ yields spatial weighted auto-covariance as follows”.

**[Reviewer #1]** Page 6: equation (9).Would this term be a correction or modification of the LISA index?

**[Reply]** No. The correction or modification of LISA based on Moran’s index is equation (33).

Equation (9) represents the general expression of the proportionality coefficient between the sum of local Moran’s indexes and the global Moran’s index. Based on this expression, it is relatively easy to see the inference error caused by Anselin's making use of row normalization spatial weight matrix.

**[Reviewer #1]** Page 6: equation (12)--check if all formulas were called in the text!

**[Reply]** The related statements have been improved now.

**[Reviewer #1]** Page 7: equation (14).

**[Reply]** Equation (14) has been made clearer, and a paragraph was added to the text to explain the problem as below:

“Looking at equation (14) alone, we may think that there is no problem. However, by summing equation (13), it is impossible to extract an independent equation (14), and this is exactly the problem.”

**[Reviewer #1]** Page 10: equation (26). Would this term be a correction or modification local Geary index?

**[Reply]** No. The correction or modification of local Geary index is equation (38).

Equation (27) represents the corrected formula of relationships between the local Geary’s coefficients and the global Geary coefficient.

The related statements in the manuscript has been improved now.

**[Reviewer #1]**Page 10: equation (26). What happens with the sample size n, large enough?

**[Reply]** There are no special requirements for sample size here. If *n* is large enough, then

$\gamma_{c}=\frac{2nV_{0}}{n-1}\to2V_{0}$ (25)

And thus

$\sum_{i=1}^{n} C_{i}^{*}=\frac{2nV_{0}\sigma^{2}}{n-1}C=\gamma_{c}\sigma^{2}C=2V_{0}\sigma^{2}C$. (26)

As we know, Moran’s index is based on population standard deviation, and the divisor is *n*, while Geary’s coefficient is base4d on sample standard deviation, and divisor is *n*-1.

**[Reviewer #1]** Page 10: equation (27). For *n* big enough the sum is *C*.

**[Reply]** In Anselin’s definition for local Geary’s coefficient, a divisor, 2, is ignored. So, when *n* is large enough, the sum of *C_i_* does not equal *C*. This defect is addressed in equation (38).

In fact, according to Anselin (1995), if *n* is large enough, and the spatial weight matrix is symmetry, then

$\sum_{i=1}^{n} C_{i}^{**}=\frac{2n}{n-1}C\to2C$. (27)

**[Reviewer #1]**Page 12: uses distance measurement, such as Euclidean distance!.

**[Reply]** The expressions have been revised now.

**[Reviewer #1]** Page 17: (**Spatial distance matrix**) (*d_ij_*)

**[Reply]** The title of Table 5 has been revised.

**[Reviewer #1]** Page 18: Figure 1, yellow

**[Reply]** The figure has been revised, and a note has been added below the figure.

**[Reviewer #1]** Page 26-27: Conclusions: do not make the text bold.

**[Reply]** The font has been modified.
